# Features of Electrochemical Hydrogen Pump Based on Irradiated Proton Exchange Membrane

**DOI:** 10.3390/membranes13110885

**Published:** 2023-11-20

**Authors:** Nataliya A. Ivanova, Boris V. Ivanov, Ruslan M. Mensharapov, Dmitry D. Spasov, Matvey V. Sinyakov, Seraphim V. Nagorny, Evgeny D. Kazakov, Petr V. Dmitryakov, Artem V. Bakirov, Sergey A. Grigoriev

**Affiliations:** 1 National Research Center “Kurchatov Institute”, 1, Akademika Kurchatova sq., 123182 Moscow, Russia; kapjicohh@gmail.com (B.V.I.); mensharapov_rm@nrcki.ru (R.M.M.); spasovdd@outlook.com (D.D.S.); mmatveimatvei4@gmail.com (M.V.S.); kazakov_ed@nrcki.ru (E.D.K.); dmitryakov_pv@nrcki.ru (P.V.D.); bakirov.artem@gmail.com (A.V.B.); grigoryevsa@mpei.ru (S.A.G.); 2 National Research University “Moscow Power Engineering Institute”, 14, Krasnokazarmennaya st., 111250 Moscow, Russia; 3 Institute of Modern Energetics and Nanotechnology, D. Mendeleev University of Chemical Technology of Russia, 9, Miusskaya Square, 125047 Moscow, Russia; nagornyy126@yandex.ru; 4 Enikolopov Institute of Synthetic Polymeric Materials of Russian Academy of Sciences, 70, Profsoyuznaya st., 117393 Moscow, Russia; 5 HySA Infrastructure Center of Competence, Faculty of Engineering, North-West University, Potchefstroom 2531, South Africa

**Keywords:** proton exchange membrane, electrochemical hydrogen pump, fusion fuel cycle, irradiated ionomer, I-V curves, membrane degradation

## Abstract

An electrochemical hydrogen pump (EHP) with a proton exchange membrane (PEM) used as part of fusion cycle systems successfully combines the processes of hydrogen extraction, purification and compression in a single device. This work comprises a novel study of the effect of ionizing radiation on the properties of the PEM as part of the EHP. Radiation exposure leads to nonspecific degradation of membranes, changes in their structure, and destruction of side and matrix chains. The findings from this work reveal that the replacement of sulfate groups in the membrane structure with carboxyl and hydrophilic groups leads to a decrease in conductivity from 0.115 to 0.103 S cm^−1^, which is reflected in halving the device performance at a temperature of 30 °C. The shift of the ionomer peak of small-angle X-ray scattering curves from 3.1 to 4.4 nm and the absence of changes in the water uptake suggested structural changes in the PEM after the irradiation. Increasing the EHP operating temperature minimized the effect of membrane irradiation on the pump performance, but enhanced membrane drying at low pressure and 50 °C, which caused a current density drop from 0.52 to 0.32 A·cm^−2^ at 0.5 V.

## 1. Introduction

Currently, the scope of application of electrochemical devices is expanding, without being limited to just hydrogen energy technologies. The use of water electrolyzers with a proton exchange membrane (PEM) for related technologies of water purification from the radioactive hydrogen isotope—tritium—in the fuel cycle of nuclear/fusion reactors is well-established. These technologies will also be used in water detritiation systems for the International Thermonuclear Experimental Reactor (ITER) reactor. Water electrolyzers act as the bottom flow circulation unit in isotope separation plants in the water-hydrogen systems based on the Combined Electrolysis and Catalytic Exchange (CECE) process [1,2]. Recently, the possibility of using an electrochemical hydrogen pump (EHP) in the fuel cycle circuit for extracting, purifying and compressing hydrogen (including all isotopes) in the gas phase has also been growing in popularity [3,4,5]. However, only EHPs based on solid oxide electrolytes have been studied for application in this area [6,7,8]. The main disadvantage of such devices is low current densities (less than 10 mA·cm^−2^) and hence low efficiency and high power consumption [7]. The use of PEM can provide a significantly higher proton conductivity and current densities of about 1 A·cm^−2^ [9].

The use of electrochemical devices under new operating conditions requires additional research for their adaptation [10]. From the viewpoint of operating parameters in the fuel cycle of a fusion reactor, the resistance of systems to ionizing radiation becomes a defining factor. Radiation exposure leads to various degradation processes in polymers and often to an irreversible decrease in their mechanical and physicochemical properties [11,12,13,14], which may entail dangerous consequences under the conditions of a fusion facility. The radiation can lead to the formation of additional cross-links between polymer chains [15,16,17,18]. Additionally to ionizing radiation, tritium decay leads to helium (decay product) accumulation in the membrane volume, which can cause the formation of helium bubbles in the membrane volume and disruption of the PEM structure during long-term operation [19].

Materials of devices for the fusion fuel cycle may be exposed to different types of radiation: ß radiation from tritium (in the gaseous state and/or in the form of water) and other isotopes de-cay, γ radiation from neutron-activated materials and bremsstrahlung X-ray [18,20,21,22], causing a gradual change in their structure and properties. Some features of the effect of various types of radiation, including the dependence on the radiation dose and conditions, are presented in [23,24,25,26,27]. Electron beam accelerators [28], X-rays [29], Co-60 (γ-radiation) [14,23,25,29,30,31,32] and impregnation with tritiated water (ß-radiation) [18,21,32] are used as radiation sources in the studies. Both the membrane/ionomer solution taken separately [22,33,34,35,36] and the electrochemical cell as a whole are exposed to irradiation [13]. The effective radiation dose ranges from approximately a few Gy to thousands of kGy, and the radiation energy ranges from tens of keV to tens of MeV, which is equivalent to a time span from several days to 5 years of exposure to tritium under conditions of the nuclear/fusion reactor fuel cycle [21,28]. The irradiation is carried out in a vacuum, in air, in an inert gas or hydrogen environment, in the presence of water in the temperature range from room temperature to ~100 °C. Despite such a diversity of research conditions and the absence of any systematic assessment in the literature, most of the obtained results fit into the general patterns of degradation processes in membranes under the influence of ionizing radiation. 

The processes of membrane degradation under the influence of ionizing radiation can be divided into two groups: cleavage of the ether bond at the end of the chain with the formation of polymer radicals, and cleavage of the ester bond near the main perfluorinated chain and of the C-F bond by radicals. The degradation process of the first type can be caused by direct interaction between radiation and the membrane; the second type is associated to a greater extent with the formation of hydroxyl and peroxyl radicals because of the radiolysis of water. At low doses of radiation, the effect of cross-linking of matrix chains is also observed, but with increasing radiation dose it is leveled out. Degradation processes in membranes exposed to ionizing radiation are generally nonspecific and occur through four main mechanisms [11] of the interaction of hydroxyl OH* and peroxyl radicals H_2_O_2_* with the structure of the polymer molecule, namely with the groups -COOH, C-S, C-O-C, C-F. As a result of irradiation, fragments of chains, both side and matrix, are detached and released from the membrane (with the formation of sulfate and fluoride ions, respectively), often leading to an increase in the C/S ratio [20,28,29]. Moreover, in the studies, the yield of fluoride ions from the membrane exceeds the yield of sulfate ions (total organic carbon to total inorganic carbon content or TOC:TIC) in the range of 2–10 times (depending on the type of radiation and the effective dose received), which indicates the predominance of mechanisms of membrane destruction due to the interaction of radicals directly with the C-F groups of the hydrophobic matrix -(CF_2_)_n_-. This effect is typical for all considered types of radiation, received doses and environments in which the membrane was placed during the irradiation. In [20], in the presence of hydrogen the release of sulfate ions into an aqueous solution during the membrane irradiation practically stops, while the concentration of fluoride ions remains unchanged. The separation and release of chain fragments from the membrane volume leads to a decrease in its equivalent mass: the equivalent mass of the membrane at an absorbed dose of 50 kGy decreased by 20% compared to the initial value [24,36].

The destruction of hydrophobic clusters in the membrane structure leads to a deterioration of the physicochemical and mechanical properties of membranes, including strength and elasticity. Most researchers agree that even with a small dose of radiation, losses in the elasticity of perfluorosulfonic acid (PFSA) membranes can reach several orders of magnitude in stress-strain tests [28]. A sharp drop in mechanical properties is observed at small doses of exposure, but by 500 kGy it gradually reaches a plateau [30], which is explained by the destruction of the crystallite structure followed by stabilization of the amorphous structure of the membranes. 

At the same time, stability and even growth of some membrane characteristics are observed during irradiation, even at high doses; these are such characteristics as gas permeability, ionic conductivity and water uptake, which play an important role in electrochemical properties of the membrane [14,18,23,24,25,30,31,36]. Proton conduction of polymer membranes is an electrochemical characteristic that largely determines the efficiency of devices on their basis. Proton conduction depends on the formation of proton-conducting channels in the membrane structure with hydrophilic -SO_3_H groups in the center of the channel. The detachment of side chains and the release of -SO_3_H from the membrane volume into the solution lead to a decrease in the efficiency of proton conduction and an increase in the ohmic resistance of membranes. However, in accordance with the degradation mechanisms [11], the breaking of carbon chains under irradiation leads to the formation of new acidic groups -COOH as the final product of the main and side reactions. Substitution, including excess one (due to the rupture of the C-F groups of the matrix), of -SO_3_H with -COOH groups allows maintaining the ion exchange capacity and water uptake of irradiated membranes within the range characteristic of non-irradiated samples. 

Despite the differences when using different irradiation sources, general trends remain the same both for the use of directly tritiated water and electron is beams and for the simulation of irradiation conditions similar to tritium with X-rays or gamma radiation. In the case of using beta radiation, the degradation effects are less pronounced compared to the use of gamma radiation, the difference being about 20%, with similar patterns of changes in characteristics [30]. 

From the point of view of studying irradiated membranes directly as part of an electrochemical cell (electrolyzer or fuel cell), the data presented in open sources are limited to a few works, and the results presented therein are contradictory. In the studies of the effect of ionizing radiation on components of a fuel cell with a PEM, the efficiency of the fuel cell decreased with increasing irradiation time, and by 200 s it was about 50% of the initial one, with the cell as a whole exposed to irradiation [24]. Despite the decrease in the equivalent mass of the membrane itself leading to an increase in its proton conductivity, the observed decrease in the fuel cell performance was attributed to a deterioration in the interaction between the ionomer and catalyst due to the radiation exposure. Studies of irradiated membranes in electrolytic cells (membranes were irradiated separately) showed the absence of any influence of radiation on the operation of the device up to doses of more than 1000 kGy. The water electrolyzer performance coincided with the error limits both when using irradiated membranes and when comparing them with a non-irradiated sample [20,21]. The differences in the results obtained can be caused both by the differences in irradiation techniques (cell as a whole versus separate membrane) and by the influence of radiation on the ongoing electrochemical processes (fuel cell versus electrolyzer). There are no similar studies for the PEM-based EHP, and since the EHP is a combination of reactions with the hydrogen of the fuel cell and water electrolyzer, it is premature to draw generalized conclusions about the effect of an irradiated membrane on the EHP efficiency.

In this work, we studied the effect of ionizing radiation on the structural properties of the PEM and the efficiency of the EHP with the irradiated membrane. The performance of the EHP was investigated in the temperature range of 30–50 °C and at pressures of 0.03 and 0.1 MPa, where subatmospheric pressures in the system are associated with ensuring safety in case the hydrogen isotope—tritium is used. The study provides new results on the possibility of using highly efficient EHP with PEM in the fusion fuel cycle.

## 2. Materials and Methods

### 2.1. Materials

#### 2.1.1. Membrane Preparation

The 150 μm thick Aquivion^®^ E98-15S membrane was used as the test sample. Before the study, the membranes were converted to the H^+^ form according to the procedure described in [37]. The membrane was irradiated in an RS-20MR installation [38] with an electron beam current of 75 kA and an electron energy of 1–1.5 MeV. The pulse duration was 500 ns. Due to the location of the membrane behind the anode of the RS-20MR installation, its irradiation was carried out bremsstrahlung X-ray radiation that was formed from an electron beam with an average energy of 1 MeV and scattered electrons with energies from 100 to 300 keV. The absorbed dose was 5 ± 1 Gy, it was calculated based on the measurements of the thermoluminescent dosimeter. Irradiation was carried out on saturated liquid water membranes.

An absorbed dose of 5 Gy corresponds to several hours of operation with tritium deuterium mixture for primary fuel separation or several days for secondary fuel separation—purification.

This dose is enough to observe the initial degradation process in the membrane structure and properties.

#### 2.1.2. Membrane Electrode Assembly (MEA) Preparation

The assembly of the EHP cell MEA was carried out in the same way as in the work [39]. The anode comprised a gas diffusion layer (GDL)—hydrophobic carbon paper of Sigracet 39 BC brand (with a microporous hydrophobic layer on one side), a catalytic layer (CL)—hydrophobic electrocatalyst Pt/C (platinum content 40 wt.%, support carbon black of Vulcan XC-72 grade, Teflon as a support water repellent, with the water repellent content in the support of 10 wt.%). Hydrophobic properties of the anode are determined by the fusion fuel cycle requirements, namely the dry inlet gas. The cathode: GDL—domestically produced hydrophilic carbon paper, CL—hydrophilic Pt/C electrocatalyst (platinum content 40 wt.%, support carbon black of Vulcan XC-72 grade). The hydrophilicity of the cathode ensures adequate water management. The electrode area of the MEA was 7 cm^2^, and the loading of the anode and cathode CL was 0.8 mg·cm^−2^.

### 2.2. Methods

#### 2.2.1. Water Uptake

The prepared membranes were dried in a vacuum oven at a temperature of 110 °C. Dry membrane samples were immersed in deionized water for 24 h at room temperature. The mass of the samples was measured before and after the immersion. The water uptake was calculated according to the formula:

(1) $$Water\;uptake\;\left(\%\right)=\frac{m_{1}-m_{0}}{m_{0}}\times 100\%$$

where *m*_0_ and *m*_1_ are e masses of the dry and wet membrane, respectively. The water uptake measurements were taken at least three times for each sample.

#### 2.2.2. Membrane Conductivity

The measurements were carried out for membrane samples soaked in deionized water at room temperature. The in-plane proton conductivity of the membranes was measured using a home-made two-electrode conductivity cell, made of Teflon^®^, with platinum electrodes, and a CorrTest CS350 electrochemical station (CorrTest Instruments, Wuhan, China) with an electrochemical impedance spectroscopy (EIS) module. The frequency range was 0.1–10^6^ Hz, the amplitude of alternating potential was 20 mV, and the constant potential was 0 V. Experimental impedance data were fitted with an equivalent circuit (*R_m_*, *CPE_m_*) (*R_i_*, *CPE_i_*), where the parallel combination of resistor *R_m_* and constant phase element *CPE_m_* represents the membrane impedance, and the parallel combination of *R_i_* and *CPE_i_* describes the interfacial impedance, which is affected by the roughness of the membrane/electrode surface and the dimensional characteristics of the electrodes [40]. The specific resistivity (*ρ*) and conductivity (*σ*) of the membrane were calculated according to the expression:

(2) $$\rho =\frac{1}{\sigma }=\frac{R_{m}\cdot h\cdot \delta }{L}$$

where *h* is the width of the membrane (*h* = 1.5 cm), *δ* is the thickness of the membrane (*δ* = 150 μm), and *L* is the distance between two electrodes (*L* = 2 cm).

#### 2.2.3. Small-Angle X-ray Scattering (SAXS)

High-resolution small-angle diffraction patterns of Aquivion^®^ membranes were recorded with a S3-Micropix SAXS camera manufactured by Hecus (Cu Kα, λ = 1.542 Å). Two detectors were used: a two-dimensional Pilatus 100 K and a PSD 50 M linear position-sensitive detector operating at a pressure of 8 bar Ar/Me. A Xenocs Genix generator supplied high voltage (50 kV) and current (1 mA) for the detectors. To eliminate the influence of air, the X-ray optics system and the camera were evacuated to a pressure of (2–3) × 10^−2^ mmHg. 

#### 2.2.4. Thermogravimetric Analysis (TGA)

The thermal stability of the samples was studied with a combined MettlerToledo TGA/DSC3+ thermal analyzer in a dynamic mode in the temperature range from 30 to 700 °C in a nitrogen flow (99.999%) of 50 mL/min at a heating rate of 10 °C/min. Standard open ceramic crucibles with a volume of 75 μL were used. The temperature determination accuracy is 0.1 °C. The scale accuracy is up to 0.001 mg.

#### 2.2.5. Electrochemical Studies of the EHP Cell

The procedure for recording I-V curves and a description of the experimental setup were presented in detail in previous works [8,39]. The cell temperature was set and controlled using a water circulation thermostat. To obtain parameters of the EHP cell from electrochemical data and evaluate the effects of ionizing radiation and temperature, I-V curves were analyzed using the following equations [8]:

(3) $$E_{cell}=E_{Nernts}+\eta_{Activation}+\eta_{Ohmic}+\eta_{Mass\;transfer}$$

where *E_Cell_* is the EHP cell voltage, *E_Nernst_* is the Nernst potential, *η_Activation_* is the activation overpotential, *η_Ohmic_* is the ohmic overpotential, and *η_Mass transfer_* is the mass transfer overpotential. The value of the Nernst voltage depends on the hydrogen pressure at the EHP anode (*P_Anode_*) and cathode (*P_Cathode_*) and is determined by the expression:

(4) $$E_{Nernts}=\frac{RT}{nF}ln\left(\frac{P_{Cathode}}{P_{Anode}}\right)$$

where *R* is the universal gas constant (*R* = 8.314 J/(mol K)), *T* is the EHP temperature, n is the number of electrons transferred per hydrogen molecule (*n* = 2), and *F* is the Faraday constant (*F* = 96.485 C·mol^−1^) [41].

Since the reaction of oxidation and hydrogen evolution on a platinum electrocatalyst occurs at a high rate, the contribution of activation losses to the overpotential of the EHP can be neglected [42]. Ohmic overpotential is determined by Ohm’s law according to the expression [43,44]:

(5) $$\eta_{Ohmic}=iR_{EHP}$$

where *i* is the output current of the EHP, and *R_EHP_* is the ohmic resistance of the EHP components, the largest contribution to which is made by the membrane resistance. According to the Mendeleev–Clapeyron equation, Faraday’s law and the Fick equation, the expression for the mass transfer overpotential can be represented as:

(6) $$\eta_{Mass\;transfer}\;=\;\frac{RT}{2F}\left(ln\left(1-\frac{i}{i_{lim,Anode}}\right)+ln\left(1-\frac{i}{i_{lim,Cathode}}\right)\right)$$

where *i_lim_* is the limiting current. When the same material is used for the cathode and the anode, the expression for the cathode limiting current is related to the anode limiting current by the expression:

(7) $$i_{lim,Cathode}=i_{lim,Anode}\frac{P_{Cathode}}{P_{Anode}}$$

The fitting was carried out using an algorithm written in Python 3.10 and the *curve_fit* function of the SciPy software package, which is an implementation of the nonlinear least squares method. The value of the ohmic resistance was limited from above based on the value obtained by linear approximation of the initial region of the I-V curve (up to 0.1 V).

## 3. Results and Discussion

### 3.1. Water Uptake

Table 1 shows the membrane water uptake values. Irradiation of the membrane, within the error limits, did not affect the water uptake at room temperature. This result is associated with the replacement of -SO_3_H with hydrophilic groups -OH, and -COOH because of degradation processes in the membrane.

### 3.2. Membrane Conductivity

**Figure 1:**
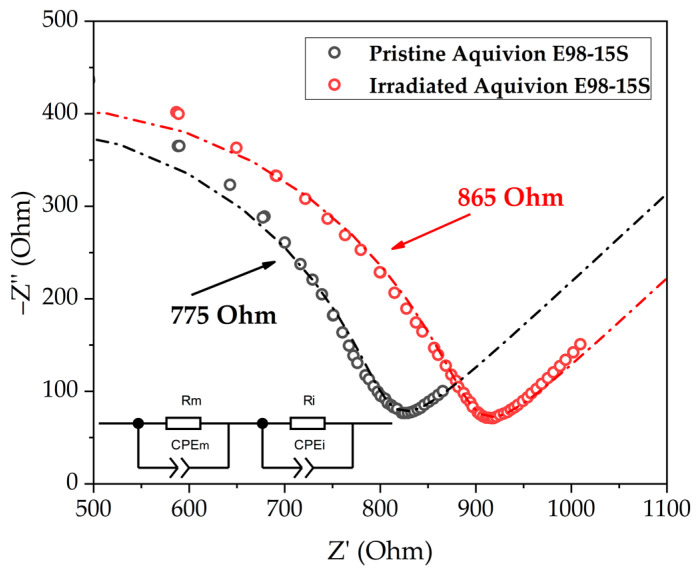
Nyquist plot of two membranes: Aquivion^®^ E98-15S (standard) and Aquivion^®^ E98-15S (irradiated) and the fitting (dash-dotted line) with the equivalent circuit (inset).

Figure 1 shows the Nyquist plot for the studied membranes. After irradiation, an increase in the membrane resistance by 12% is observed at room temperature. The increase in the resistance may indicate degradation of the membrane and detachment of part of the -SO_3_H because of the exposure to ionizing radiation.

Table 2 shows values of the volumetric resistivity and conductivity obtained for membranes in contact with water. The difference with the literature data can be explained by the impedance measurement method and the preparation of the membranes; however, a comparative analysis within this study is possible.

### 3.3. Small-Angle X-ray Scattering

**Figure 2:**
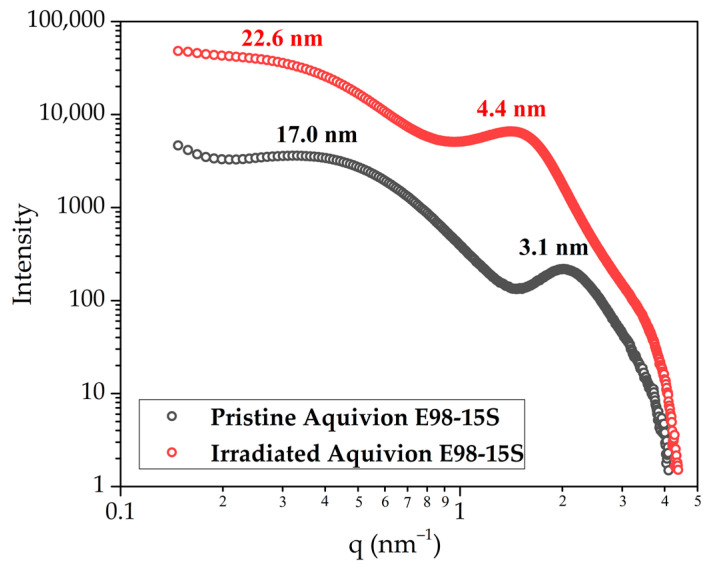
SAXS curves of Aquivion^®^ E98-15S membranes before and after irradiation.

Figure 2 shows SAXS curves for samples of the standard Aquivion^®^ E98-15S membrane and the membrane after irradiation. The irradiation resulted in an increase in the distance between the lamellar domains (from 17.0 nm to 22.6 nm) and the ion channels of the membrane (from 3.1 to 4.4 nm).

According to a number of works [46,47], an increase in the distance between the neighboring lamellar domain and ion channel structures corresponds to an increase in the moisture content of the membrane, however, in the case of the irradiated membrane, no increase in water uptake was observed. Since during membrane irradiation, there occurs a partial loss of -SO_3_H, the appearance of breaks in the perfluorinated polymer, and the formation of hydroxyl and carboxyl groups, reorganization of the hydrophobic backbone is possible. As a result of structural changes as obtained from the SAXS data, the distance between neighboring acidic groups can increase, leading to an increase in the characteristic size of lamellar structures, while the size of ion channels, despite the degradation of -SO_3_H, changes only slightly due to the appearance of new hydrophilic and acidic groups [22,33]. Thus, these structural changes, due to the low efficiency of proton conduction of new ionic groups, cause a decrease in the PEM conductivity and may lead to a loss of the EHP cell performance.

### 3.4. Thermogravimetric Analysis

**Figure 3:**
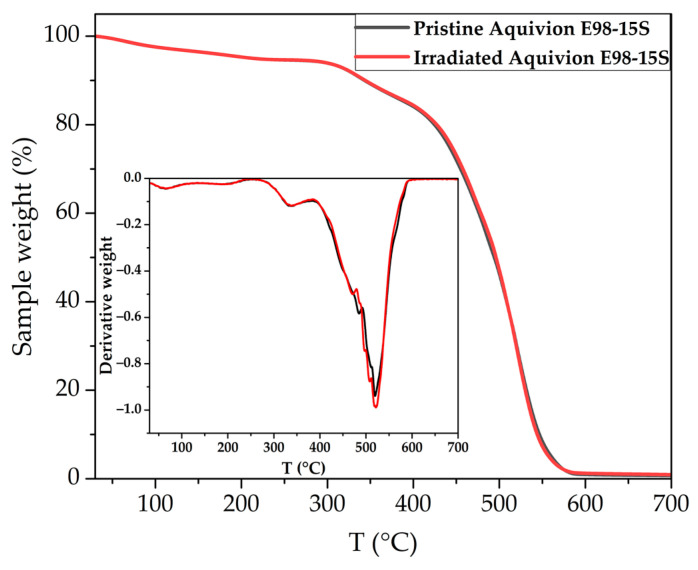
TGA and DTG (inset) graph of pristine and irradiated Aquivion^®^ E98-15S membranes.

The TGA data are presented in Figure 3, where the inset graph shows the first derivative of the TGA curve (DTG). There were no significant changes in the thermal stability of the membrane during irradiation. The figure also shows that there is no change in the position of the peak in the temperature range of 280–380 °C corresponding to the decomposition of -SO_3_H [28,48]. In the temperature range of 380–590 °C, a multi-stage reaction of decomposition of CF_2_–CF_2_ polymer chains occurs, the position of the main peak also did not change after irradiation of the membrane; however, there is a shift of the pronounced peak in the region of 470–485 °C, which may characterize deterioration of the thermal stability of the perfluorinated polymer due to the backbone degradation. Thus, in the operating temperature range of the EHP, irradiation does not affect the thermal stability of the Aquivion^®^ E98-15S membrane. Therefore, the operating temperature of the EHP can be increased to improve the electrochemical performance of the cell.

### 3.5. Electrochemical Studies of the EHP Cell

**Figure 4:**
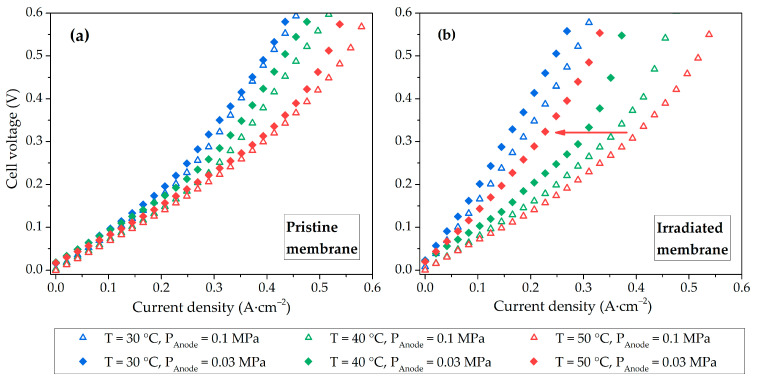
I-V curves of EHP cell with pristine (**a**) and irradiated (**b**) membranes.

For the pristine membrane, the influence of hydrogen pressure at the anode does not have a significant effect on the EHP efficiency; with increasing temperature, the current increases because of the improved electrocatalyst activity and membrane conductivity (Figure 4a). For the irradiated membrane, the effect of pressure at the anode and temperature is more pronounced because of water balance disturbance in the membrane volume (Figure 4b). At a temperature of 50 °C and a pressure of 0.03 MPa, the efficiency of the EHP cell with the irradiated membrane decreases due to its more intense drying, which can be explained by the enhanced evaporation of water and increased vapor diffusion rate, causing dehydration of the membrane [8,49], as well as by low water retention ability of the irradiated membrane.

**Figure 5:**
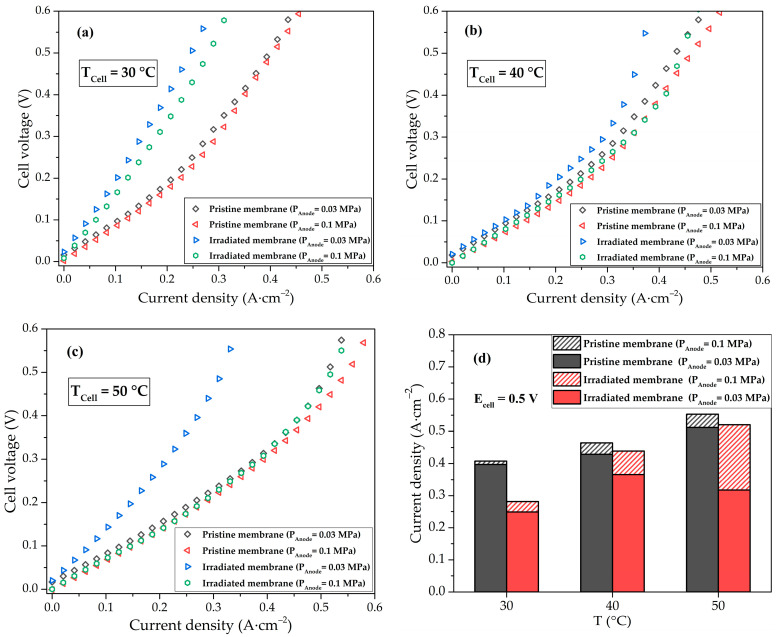
I-V curves of the EHP cell with pristine and irradiated membranes at 30 °C (**a**), 40 °C (**b**), 50 °C (**c**), and the EHP current density at the cell voltage of 0.5 V (**d**).

When comparing the I-V curves for two samples of EHP cells at different temperatures, the irradiated membrane at 30 °C demonstrates lower efficiency (Figure 5a), however, with increasing temperature, the efficiency of the two EHP cells turns out to be comparable (Figure 5b,c). This effect can be attributed to a stronger dependence of the conductivity of hydroxyl groups in the irradiated membrane on temperature in the considered range than for -SO_3_H [50]. However, at low pressure, the efficiency of the EHP cell with the irradiated membrane drops with increasing temperature due to the predominance of membrane dehydration. Figure 5d shows the current density of the EHP cell at 0.5 V. The efficiencies of the EHPs with irradiated and pristine membranes are close at elevated temperatures and atmospheric pressure, and the difference in current densities does not exceed 5%. The minimum discrepancy in current densities for different membranes at 0.03 MPa is reached at 40 °C and equals 14%. These results determine the optimal operating conditions for the EHP cell with irradiated PEM.

**Figure 6:**
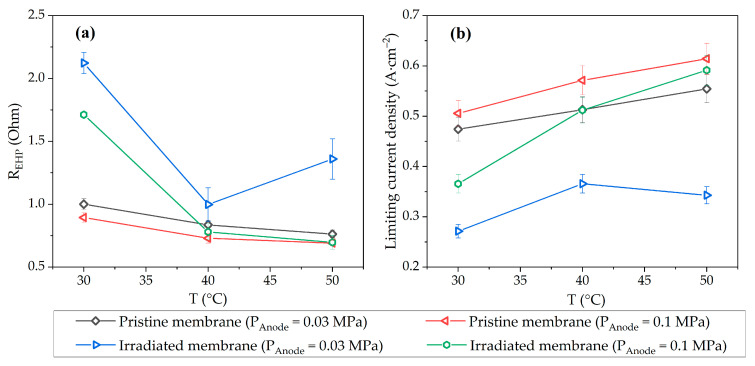
Dependence of the ohmic resistance (**a**) and limiting current density (**b**) on the temperature of the EHP cell. Parameter error bars indicate standard deviations of the fit.

As a result of the description of the experimental data with the model curve, the values of the ohmic resistance of the EHP cell and the limiting current characterizing mass transfer effects were obtained (Figure 6). At temperatures of 40 and 50 °C, the values of these parameters turn out to be almost the same within the measurement error for the original membrane for all pressures under consideration and for the irradiated membrane at a pressure of 0.1 MPa. Thus, in the case of an irradiated membrane, an increase in temperature makes it possible to minimize the effect of irradiation on the membrane conductivity associated with the replacement of part of the -SO_3_H with -OH and -COOH groups. However, at low pressure, it is necessary to stabilize the water balance of the cell to prevent drying of the membrane, in particular, by adding hydrophilic inorganic fillers into the membrane [51,52] or catalyst layer [53]. 

The limiting current densities shown in Figure 6b follow the opposite trend of resistance as a function of temperature. This relation indicates that in this case, the limiting current depends to a greater extent on the membrane properties, namely the number of open water channels in the PEM volume, than on hydrogen transport through porous electrode media.

The obtained results agree with previously published data and explain their inconsistency. In [36], a twofold decrease in the characteristics of fuel cells at the operating temperature of 30 °C is observed for the irradiated PEM, while at the same time, when the electrolyzer operates at 60 °C, no drop in the efficiency is observed for the irradiated membrane [21]. This effect characterizes the structure of the membrane after irradiation, namely the detachment of -SO_3_H groups from the side chains and the formation of -OH and -COOH groups instead, which is also consistent with the obtained structural data. In this case, there is a pronounced dependence of the conductive properties of the membrane on temperature (in the range of 20–60 °C), which is reflected in the EHP performance. In the case of an electrolysis cell, the effect of reducing the water uptake of the irradiated membrane is also less pronounced compared to the fuel cell.

## 4. Conclusions

The use of EHP with PEM as part of fusion fuel cycle systems is an effective way to extract, concentrate and compress hydrogen, including all of its isotopes, in one stage in a single device. Due to the radioactivity of tritium, an important parameter of EHP components is resistance to ionizing radiation. In this work, the structural properties and electrochemical performance of the irradiated membrane as a key element of the EHP with PEM were studied for the first time. A sample of the Aquivion^®^ E98-15S membrane was exposed to a bremsstrahlung X-ray radiation, the absorbed dose was 5 ± 1 Gy. It was demonstrated that irradiation did not affect water uptake of the membrane, but the SAXS data showed an increase in the characteristic distance between lamellar domains (from 17.0 nm to 22.6 nm) and between ion channels (from 3.1 to 4.4 nm), which was attributed to the detachment of the -SO_3_H groups, formation of -OH and -COOH groups, and reorganization of the PEM structure. The lower conductivity of the irradiated membrane of 0.103 S·cm^−2^ compared to the pristine membrane conductivity of 0.115 S·cm^−2^ indicated a lower efficiency of proton conduction of the new groups. The TGA analysis data for the PEM after irradiation showed no changes in thermal stability, so the operating temperature of the irradiated EHP can be increased to improve the electrochemical performance of the cell. To investigate the performance of the membrane as a part of the EHP cell, I-V curves were recorded at various operating temperatures and anode gas pressures. It was shown that the efficiency of the EHP with the irradiated and pristine membranes was close at elevated temperatures and atmospheric pressure, the difference in current densities at 0.5 V did not exceed 5%, but at a pressure of 0.03 MPa the efficiency of the EHP cell with an irradiated membrane significantly decreased because of its lower water retention ability and more intense drying. In particular, the drop in the performance for the irradiated membrane was about 40% compared to the pristine membrane at 50 °C, and the minimum discrepancy in current densities was 14% at 40 °C. Thus, the results obtained showed the possibility of using EHP with PEM under conditions of tritium ionization radiation exposure and various operating conditions, and pave the way for future development of highly efficient electrochemical pump technology for the fusion fuel cycle.

## Figures and Tables

**Table 1:** Water uptake values.

Membrane	Water Uptake at 20 °C, wt. %
Aquivion^®^ E98-15S	25.0 ± 1.3
Aquivion^®^ E98-15S [45]	24.2
Aquivion^®^ E98-15S irradiated	24.3 ± 1.2

**Table 2:** Values of resistivity (*ρ*) and conductivity (*σ*) of Aquivion^®^ membranes.

Membrane	*ρ*, Ohm·cm	*σ*, S·cm^−1^
Aquivion^®^ E98-15S	8.7	0.115
Aquivion^®^ E98-05S [45]	6.7	0.149
Aquivion^®^ E98-15S (irradiated)	9.7	0.103

## Data Availability

The data presented in this study are available on request from the corresponding author.
